# Ultrasound-assisted assembly of *Auricularia auricula-jud*ae polysaccharide–soy protein isolate nanoparticles: The effect of extraction methods on functional properties

**DOI:** 10.1016/j.fochx.2026.104488

**Published:** 2026-09-16

**Authors:** Qiong Wu, Jiaxiu Wei, Shuang Li, JunXiao Du, ZhiTong Cai, TianQi Zhang, YongGang Dai

**Affiliations:** a Changchun University, Nanguan District, Changchun, Jilin Province 130022, China; b Changchun Airen Pharmaceutical Technology Co., Ltd, Changchun, Jilin Province 130000, China; c Jilin Academy of Agricultural Sciences, Nanguan District, Changchun, Jilin Province 130033, China

**Keywords:** Ultra-high-pressure extraction, Auricularia auricula-judae polysaccharides, Structural regulation, Ultrasound-assisted self-assembly, Polysaccharide–protein nanoparticles

## Abstract

This study compared the emulsifying properties and in vitro anti-inflammatory activity of black fungus polysaccharides (AAP) extracted by ultra-high-pressure-assisted extraction (UHP) and hot water extraction (HWE). UHP-AAP exhibited improved emulsifying performance, with an emulsifying activity index (EAI) of 0.62 m^2^/g and emulsifying stability index (ESI) of 79.85 min, together with enhanced anti-inflammatory activity. Using UHP-AAP and soy protein isolate (SPI), composite nanoparticles (A-S NPs) were fabricated via ultrasound-assisted self-assembly. The optimized A-S NPs (AAP/SPI = 1:1, 300 W, 10 min) showed a particle size of 318 nm, a ζ-potential of −43 mV, and a PDI of 0.2136. Hydrogen bonding, electrostatic attraction, and hydrophobic interactions promoted molecular rearrangement and network formation, improving colloidal and thermal stability. These nanoparticles possess good thermal stability and dispersibility, making them suitable as functional food carriers.

## Highlights

• Ultra-high-pressure-assisted hot water extraction(UHP) extraction reduced the molecular weight of AAP and optimized its microstructure, thereby effectively improving its emulsifying properties and anti-inflammatory activity compared with conventional hot water extraction(HWE).
• 2.Used an ultrasound-assisted non-thermal self-assembly strategy to construct black fungus polysaccharide–soy protein isolate composite nanoparticles, no chemical crosslinking agents are required, and an optimal processed of 1:1 ratio, 300 W, for 10 min is established.
• Ultrasonic-assisted self-assembly successfully fabricated uniform AAP-SPI nanoparticles driven by synergistic hydrogen bonding and hydrophobic interactions. Ultrasonic assembly induced structural rearrangement and reduced crystallinity of composites, providing explicit structural basis for nanoparticle formation.

## Graphical abstract

Created with BioGDP.com

Jiang S, Li H, Zhang L, et al. Generic Diagramming Platform (GDP): a comprehensive database of high-quality biomedical graphics. Nucleic Acids Res. 2025;53(D1):D1670-D1676. doi:https://doi.org/10.1093/nar/gkae973

**Figure:**
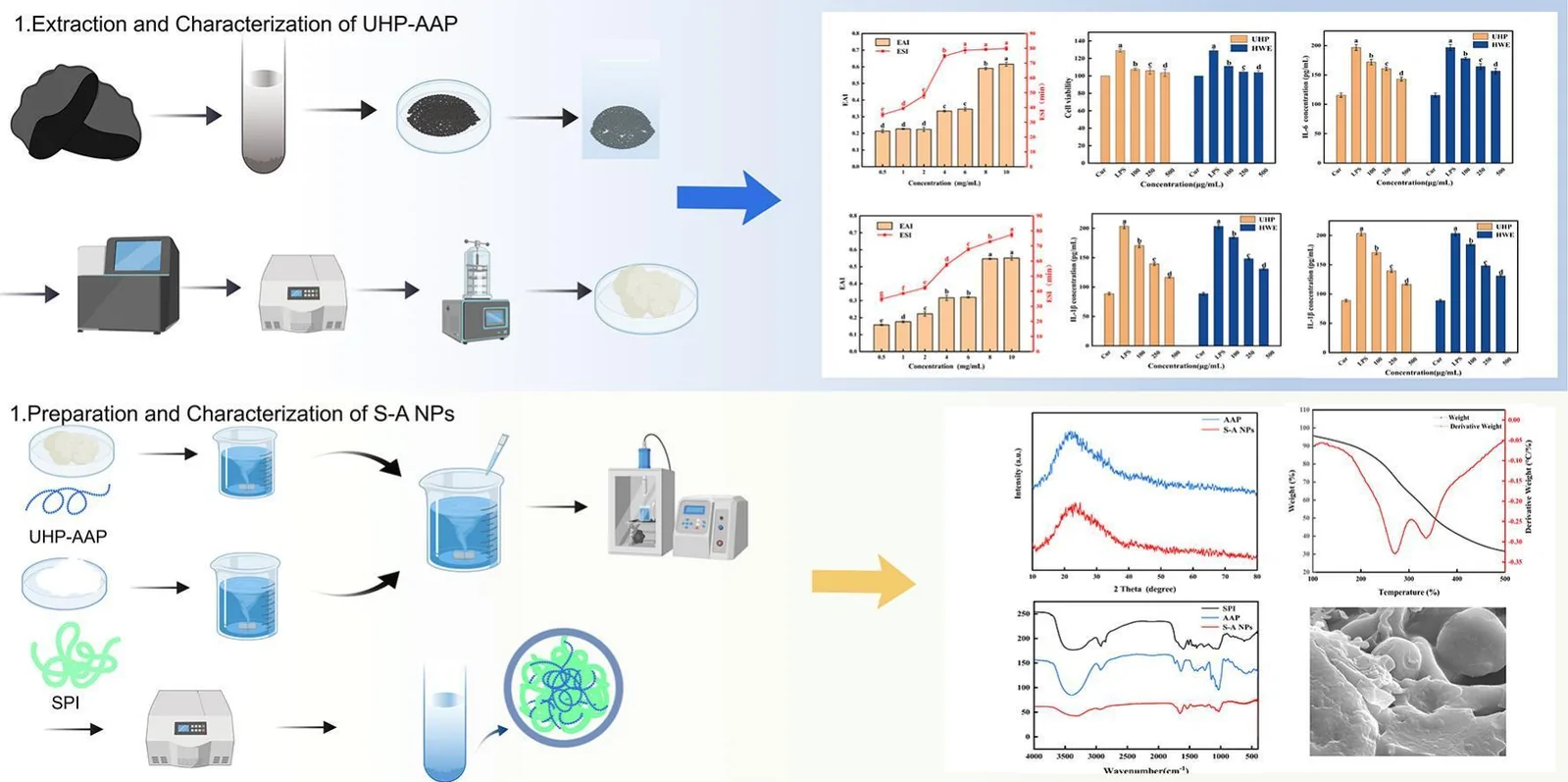

## 1 Introduction

*Auricularia auricula-judae,* commonly known as black fungus, is an edible mushroom widely consumed worldwide due to its nutritional value and diverse biological activities. It is widely distributed and cultivated in East Asia, especially in China. *Auricularia auricula-judae* polysaccharides (AAP) are a class of water-soluble natural glucans derived from the edible and medicinal fungus Auricularia auricula-judae. They possess excellent antioxidant properties, immunomodulatory activity, and biocompatibility, and hold great potential for application in the fields of functional foods and biomaterials(Fei et al., 2025; Fu et al., 2022; Xu et al., 2016). Soy protein isolate (SPI) is a major plant-derived protein widely used in food systems due to its high nutritional value, excellent functional properties, and good biocompatibility. As a plant-derived protein, SPI possesses excellent emulsifying, gelling, and self-assembling properties and has been widely used to construct delivery systems for bioactive substances(Tabatabaei et al., 2024). SPI contains abundant hydrophilic and hydrophobic amino acid residues, enabling it to undergo spontaneous structural unfolding, intermolecular rearrangement, and non-covalent cross-linking with polysaccharides under physical induction. This unique structural characteristic facilitates the stable formation of uniform nano-scale assemblies. Because AAP and SPI are complementary in terms of their structural and functional characteristics, they were selected as building blocks. AAP provide hydroxyl-rich, bioactive polysaccharide chains with immunomodulatory potential, while SPI offers excellent emulsifying capacity, food compatibility, and structural flexibility. These two biopolymers interact through hydrogen bonding, electrostatic attraction, and hydrophobic interactions, promoting the formation of stable polysaccharide-protein nanoparticles.

Ultrasound-assisted self-assembly has emerged as an effective non-thermal strategy for constructing polysaccharide–protein complexes(He, 2023). Acoustic cavitation generates localized shear forces and transient microenvironments, which may induce conformational changes(Yu, Cheng, et al., 2024). Acoustic cavitation generated by ultrasound promotes partial unfolding of protein structures under mild processing conditions, thereby exposing functional groups such as hydroxyl and carboxyl groups, as well as hydrophobic domains within protein structures, thereby exposing functional groups such as hydroxyl and carboxyl groups, as well as hydrophobic domains within protein structures. The originally dense polysaccharide clusters are also broken apart, and the tangled long chains come undone. Compared with chemical cross-linking approaches, ultrasound avoids the use of chemical reagents and provides a mild physical strategy for food-compatible nanoparticle fabrication. These structural changes facilitate intermolecular interactions, including hydrogen bonding and electrostatic attraction, ultimately driving the formation of stable polysaccharide–protein complexes(Han et al., 2022). As a result, molecular rearrangement is facilitated, contributing to the formation of uniform and stable nanoparticle systems. Compared with conventional methods such as chemical cross-linking or high-pressure homogenization, ultrasound offers advantages including reduced processing time, lower energy consumption, and the avoidance of chemical reagents, making it a promising approach for sustainable food processing applications. It is hypothesized that ultrasound treatment enhances molecular rearrangement and self-assembly. This enhanced interaction promotes efficient molecular association, resulting in nanoparticles with smaller particle size, more uniform distribution, higher absolute zeta potential, and improved colloidal stability.

To verify this hypothesis, this study compared the emulsifying properties and immunological activity of UHP-AAP and HWE-AAP. Compared with conventional HWE, UHP may regulate AAP molecular characteristics by inducing moderate chain depolymerization, reducing molecular weight, and increasing molecular accessibility. Using the superior UHP-AAP and SPI as the model system, composite nanoparticles were prepared via an ultrasonic-assisted method. The raw material ratios and ultrasonic conditions were optimized based on evaluation criteria including particle size, polydispersity index (PDI), and zeta potential. Concurrently, Fourier transform infrared spectroscopy (FT-IR), X-ray diffraction (XRD), and scanning electron microscopy (SEM) were employed to systematically analyze their structural characteristics and intermolecular interactions. This study systematically compares the effects of UHP and HWE extraction on the emulsifying and anti-inflammatory properties of AAP. Subsequently, UHP-AAP and SPI were assembled into nanoparticles through ultrasound-assisted self-assembly, systematically establishing a relationship model of “extraction method → structure → function → assembly behavior.” Unlike previous studies, which primarily focused on increasing polysaccharide yield, this study provides new insights into how extraction strategies influence the functional properties and self-assembly behavior of polysaccharides. The established correlation between extraction methods, enhanced bioactivity, and nanoparticle formation expands our understanding of the relationship between the structure and function of natural polysaccharides.

## 2 Materials and methods

### 2.1 Materials

*Auricularia auricula-judae* polysaccharides (AAP) were prepared in our laboratory from dried Auricularia auricula-judae collected in Jilin, China. The obtained AAP exhibited a glucose-dominant monosaccharide composition (91%), a degree of esterification (DE) of 76.89%, and a weight-average molecular weight (Mw) of 273.43 kDa. Soy protein isolate (SPI) was purchased from Shanxi Ruimao Biotechnology Co., Ltd. (Shanxi, China). Commercial corn oil was obtained from COFCO Fulinmen (Beijing, China). All other chemical reagents were of analytical grade and purchased from Shanghai Yuanye Biotechnology Co., Ltd. (Shanghai, China).

### 2.2 Preparation of AAP

To extract polysaccharides from black fungus using the ultra-high-pressure-assisted extraction (UHP), pretreated black fungus powder was mixed with purified water at a solid-to-liquid ratio of 1:40. The mixture was then sealed in a plastic bag suitable for ultra-high-pressure experiments. Subsequently, the mixture underwent ultra-high-pressure pretreatment at 400 MPa for 6 min. UHP treatment was performed using an ultra-high-pressure processing system (SHPP-5 L, Shanxi Lidefu Technology Co., Ltd., Shanxi, China). The treated mixture was then subjected to a water bath at 100 °C for 3.5 h(Wang, Dong, et al., 2024). After cooling to room temperature, the mixture was centrifuged to collect the supernatant. Four times the volume of anhydrous ethanol was added to the supernatant, and the mixture was allowed to precipitate in ethanol at 4 °C for 24 h. It was then centrifuged at 3500 rpm for 30 min (NX-3R, Dinghaoyuan Biotechnology Co., Ltd., Tianjin, China)., the precipitate was collected, and the ethanol was removed from the system via rotary evaporation. After vacuum freeze-drying, the dried polysaccharide was redissolved(Chen et al., 2024). Dialysis purification was performed using an ctivated dialysis bag with a molecular weight cut-off of 3500 Da. The sample was freeze-dried again to obtain UHP-assisted-extracted black fungus polysaccharide (UHP-AAP)(Liu et al., 2024).

### 2.3 Extraction of black fungus polysaccharides using the traditional hot-water method

The mixture was prepared at a solid-to-liquid ratio of 1:40 and heated in a water bath for 3.5 h, subsequent steps were the same as those described in Section 2.2, yielding black fungus polysaccharides extracted by the hot-water method (HWE-AAP).

### 2.4 Determination of the emulsifying properties of polysaccharides

UHP-AAP solutions with different mass concentrations were prepared (0.5,1,2,4,6,8,10 mg/mL). Take 20 mL of the polysaccharide solution, add 2 mL of corn oil, and homogenize for 2 min using a high-speed homogenizer at 10,000 rpm(Dai et al., 2023). Using a pipette, accurately aspirate 100 μL from the center of the homogenized emulsion into a test tube, immediately add 5 mL of a 1% SDS aqueous solution, shake well, and measure the absorbance (A) at a wavelength of 500 nm. The EAI was calculated according to the following equation based on the absorbance value (A) of black fungus polysaccharides on corn oil.

Take another 20 mL of AAP solution, add 300 μL of corn oil, and homogenize for 2 min using a high-speed homogenizer at 10,000 rpm. Using a pipette, accurately aspirate 100 μL from the center of the homogenized emulsion into a test tube, immediately add 5 mL of 1% SDS aqueous solution, shake well, and measure the absorbance A₀ at a wavelength of 500 nm. After allowing the remaining emulsion to stand for 10 min, take another 100 μL, add 5 mL of SDS, and measure the absorbance A_t. The emulsification stability index (ESI) is calculated using the following formula:

(1) $$\mathrm{ESI}=\frac{A_{0}}{A_{0}-A_{t}}$$

in the formula， t represents the standing time, which is 10 min.

### 2.5 In vitro evaluation of immunomodulatory activity

#### 2.5.1 Cytotoxicity assay

RAW264.7 macrophages in the logarithmic growth phase were digested with trypsin, and the cell concentration was adjusted to 6000 cells per well before seeding into a 96-well plate, where they were cultured for 24 h. After cell attachment, the cells were treated with UHP-AAP and HWE-AAP at different concentrations (100, 250, 500 μg/mL)(Wu et al., 2025). After 24 h, the supernatant was aspirated, and 100 μL of 10% CCK-8 solution was added. The plates were incubated for 2 h, and absorbance was measured at 450 nm. The culture medium blank served as the blank control, and the untreated cells were used as the control group. Cell viability was calculated as follows:

(2) $$\text{Cell viability}\%=\frac{A_{1}-A_{0}}{A_{2}-A_{0}}\times 100\%$$

in the formula: A1 = sample group, A0 = blank, A2 = control (untreated cells).

#### 2.5.2 Determination of nitric oxide production and inflammatory cytokines

RAW264.7 macrophages in the logarithmic growth phase were detached using trypsin. The cell suspension was seeded at 1 mL per well (cell concentration of 5.0 × 10^5^ cells/mL) into a 24-well plate. After cell attachment to the wells, the cells were treated with different concentrations (100, 250, 500 μg/mL) of UHP-AAP and HWE-AAP, as well as 1 μg/mL of LPS, using a sample-free group as the control. Culture for 24 h, collect the cell supernatant, centrifuge, and store at −80 °C for later use. Add 50 μL per well to a 96-well plate, setting up a blank control at the same time; add 50 μL of Griess I and Griess II reagents to each well in sequence, mix well, and measure the absorbance at 540 nm using a microplate reader to determine NO. The secretion levels of TNF-α, IL-1β, and IL-6 were determined using ELISA kits.

### 2.6 Ultrasonic preparation of soy protein isolate and black fungus polysaccharide composite nanoparticles (S-A NPs)

S-A NPs were prepared using an ultrasonic-assisted method. AAP and SPI were dissolved in pure water and thoroughly hydrated. They were then mixed in various ratios (1:4, 1:2, 1:1, 2:1, 4:1), adjusted to pH 3 to promote electrostatic interactions between oppositely charged groups, and subjected to constant-temperature magnetic stirring for 4 h. Ultrasonic treatment was then performed to optimize the nanoparticle preparation process by adjusting the ultrasonic power (100, 200, 300, 400, 500 W) and treatment time (2, 5, 10, 15, 20 min)(Li et al., 2021; Wu et al., 2025). Dynamic light scattering (DLS) and zeta potential measurements were performed using a Malvern Zetasizer Nano ZSP instrument (Malvern Panalytical, UK) to determine the hydrodynamic particle size, polydispersity index (PDI). All nanoparticle suspensions were uniformly diluted with deionized water to an appropriate concentration to avoid multiple scattering effects and aggregate interference. Finally, the aqueous solution of the prepared nanoparticles was concentrated and freeze-dried.

### 2.7 Structural characterization of S-A NPs

#### 2.7.1 Fourier-transform infrared spectroscopy

The chemical functional groups and intermolecular interactions of nanoparticles were analyzed using FT-IR spectroscopy (NICOLET iS5, Thermo Fisher Scientific, USA). After thoroughly grinding the sample with dried potassium bromide, infrared absorption spectra were measured in the range of 400–4000 cm^−1^, to identify characteristic functional groups and analyze characteristic functional groups(Li et al., 2021).

#### 2.7.2 X-ray diffraction analysis

X-ray diffraction analysis was used to determine the crystallinity of nanoparticles and the ordered structure formed after self-assembly(Maqsoodi et al., 2025). Samples were prepared by gently pressing the freeze-dried nanoparticle powder onto a clean glass sample holder to form a smooth, flat surface. The XRD patterns were collected over a 2θ range of 10°–90° with a step size of 0.02° at a scanning rate of 2°/min. The measurements were performed using Cu Kα radiation (λ = 0.15418 nm) operated at a tube voltage of 30 kV and a tube current of 10 mA (Sarkar & Kalita, 2022).

#### 2.7.3 Thermogravimetric analysis

Evaluates the thermal stability and decomposition characteristics of nanoparticles during heating(Yang et al., 2025). TGA was performed to evaluate the thermal stability of the assembled nanoparticles. Place the dried polysaccharide sample in an alumina crucible. Conduct thermogravimetric analysis using 99.99% high-purity nitrogen gas as the carrier gas. Increase the temperature from 100 °C to 500 °C at a heating rate of 20 °C/min(Wang et al., 2020).

#### 2.7.4 Microstructural analysis (SEM)

The microstructure of the nanoparticles was examined using a scanning electron microscope (SEM). An appropriate amount of sample powder was weighed and evenly spread onto a conductive adhesive. After gold coating, the sample was placed in the instrument to observe the microstructure of the S-A NPs(Wang et al., 2026). SEM images were collected at an accelerating voltage of 5 kV.

### 2.8 Statistical analysis

All experiments were repeated three times. The experimental results are presented as mean ± standard deviation. Graphs were generated using Origin software. Statistical analysis of the experimental data was performed using SPSS software; a statistically significant difference was defined as *p* ≤ 0.05.

## 3 Analysis of results

### 3.1 Determination of the emulsifying properties of polysaccharides

**Fig. 1:**
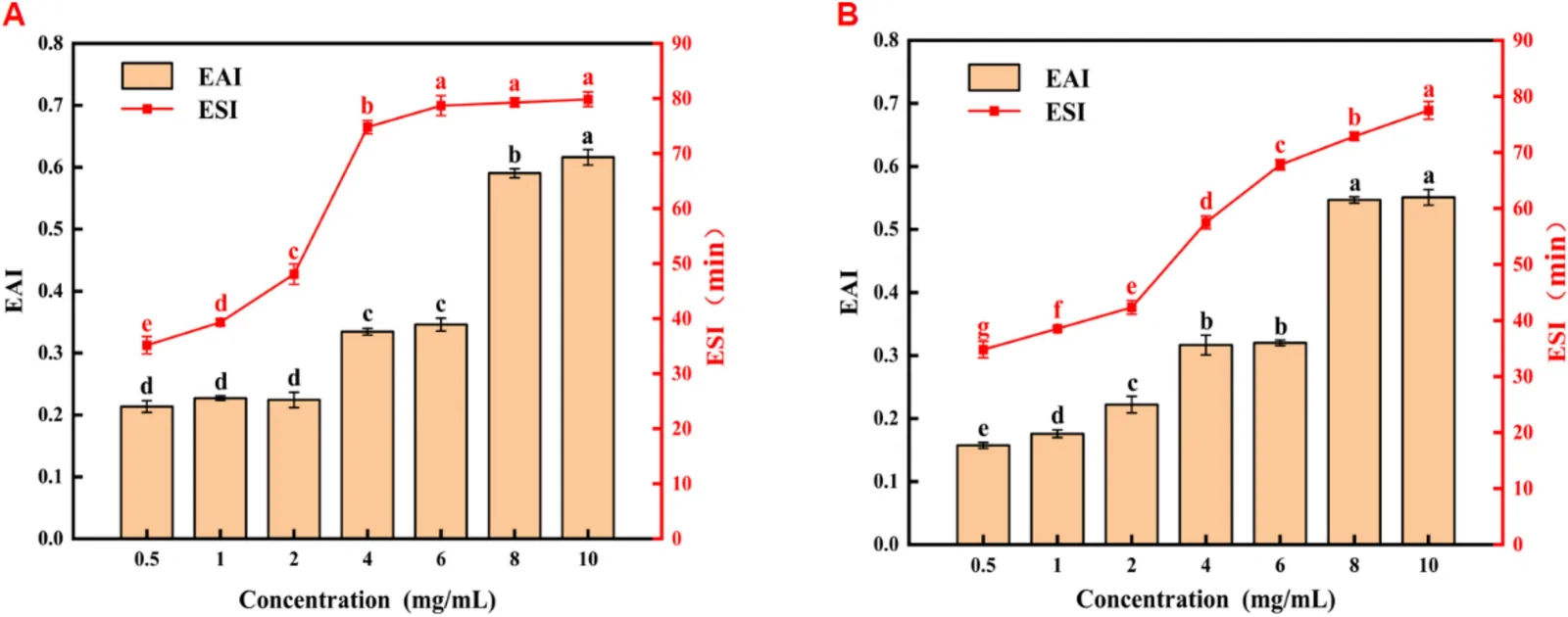
Comparison of the emulsifying properties of black fungus polysaccharides extracted using UHP (A) and HWE (B).

As shown in Figs. 1A and B, both the emulsifying activity index (EAI) and emulsifying stability index (ESI) of UHP-AAP and HWE-AAP increased gradually with rising concentration. However, there are differences between the two in terms of overall levels and the magnitude of change. UHP-AAP exhibited higher EAI and ESI across throughout the tested concentration range, with a more pronounced advantage at medium to high concentrations (≥4 mg/mL), suggesting improved emulsifying capacity and emulsion stability. UHP treatment induced moderate structural modification of AAP, resulting in partial chain depolymerization and a reduction in molecular weight(Ye et al., 2025). This moderate structural modification may improve the accessibility and mobility of polysaccharide chains, facilitating their diffusion and adsorption at the oil–water interface. Meanwhile, the remaining polysaccharide chains provide sufficient steric stabilization, while the increased accessibility of hydrophilic groups and the presence of uronic acid residues may contribute to enhanced interfacial interactions and electrostatic repulsion. These synergistic effects contribute to the formation of a more stable interfacial layer, thereby inhibiting droplet flocculation and coalescence and improving emulsion stability.(Lin et al., 2025). When the concentration is further increased (e.g., to 8–10 mg/mL), the improvement in emulsification performance tends to level off, which may be related to the saturation of interfacial adsorption.

### 3.2 In vitro immunomodulatory activity of AAP

**Fig. 2:**
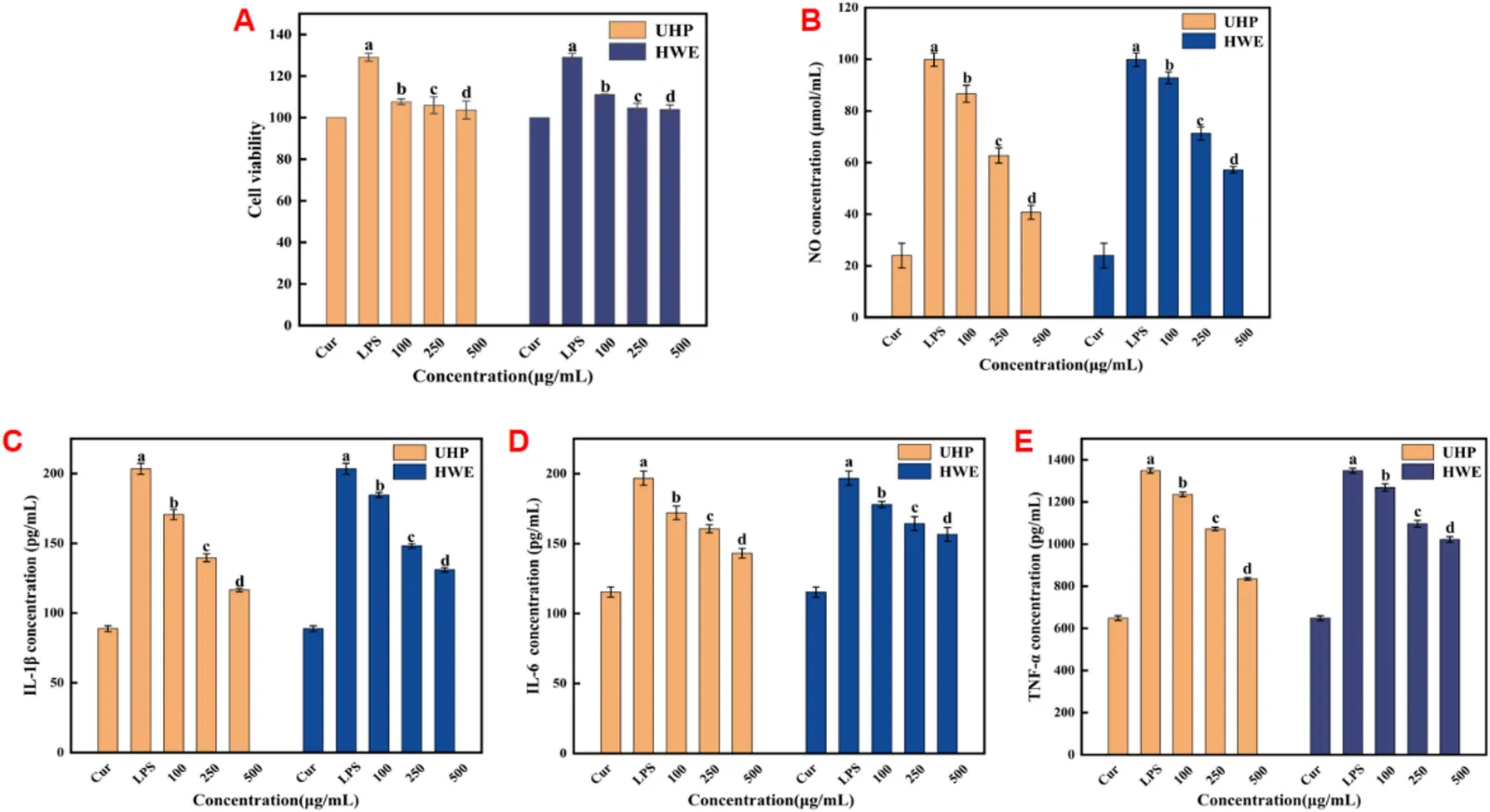
In vitro immunomodulatory activity of UHP-AAP and HWE-AAP. (A) Effects of UHP-AAP and HWE-AAP on the viability of RAW264.7 macrophages, (B) shows the effects of UHP-AAP and HWE-AAP on NO production in RAW264.7 macrophages, (C) Effects of UHP-AAP and HWE-AAP on IL-1β secretion by RAW264.7 macrophages, (D) shows the effects of UHP-AAP and HWE-AAP on IL-6 secretion by RAW264.7 cells before and after modification,(E) The effects of UHP-AAP and HWE-AAP on cytokine TNF-α secretion in RAW264.7 cells.

The immunomodulatory activity of UHP-AAP and HWE-AAP was evaluated using lipopolysaccharide (LPS)-stimulated RAW264.7 macrophages as an in vitro model (Lin et al., 2025). Cell viability, nitric oxide (NO) production, and the secretion levels of major pro-inflammatory cytokines, including interleukin-1β (IL-1β), interleukin-6 (IL-6), and tumor necrosis factor-α (TNF-α), were systematically evaluated, as shown in Fig. 2.

#### 3.2.1 Cell viability analysis

As shown in Fig. 2A, within the tested concentration range, neither UHP-AAP nor HWE-AAP exhibited significant cytotoxic effects on RAW264.7 macrophages. Cell viability remained at a high level overall, indicating that both polysaccharides possess good biocompatibility(Liu et al., 2025). Under LPS stimulation, macrophages exhibited a state of excessive activation. Both polysaccharides partially alleviated the LPS-induced excessive activation of macrophages(Guan et al., 2021). This phenomenon suggests that structural differences resulting from different extraction methods may influence the ability of polysaccharides to regulate the activation state of immune cells. UHP treatment induced moderate structural modification of AAP, including partial chain depolymerization and increased molecular accessibility, which may facilitate the exposure of bioactive groups and may facilitate interactions with immune-related cellular components(Zheng et al., 2023). The optimized molecular characteristics of UHP-AAP could contribute to more efficient modulation of macrophage activation without affecting cell viability. In contrast, the different structural features of HWE-AAP, possibly resulting from thermal-induced molecular rearrangement or degradation, may reduce the availability of functional groups, thereby weakening its immunomodulatory efficiency.3.2.2 Analysis of NO Release Levels and Cytokine Secretion.

NO, a classic marker of macrophage activation, is a central feature of the inflammatory response when produced in excess. As shown in Fig. 2B, the baseline NO release level in unstimulated RAW264.7 macrophages is extremely low, whereas LPS stimulation causes a sharp increase in NO concentration, successfully establishing an inflammatory cell model. Both UHP-AAP and HWE-AAP significantly inhibited LPS-induced excessive NO release in a concentration-dependent manner, and at all concentration gradients, the inhibitory effect of UHP-AAP was greater than that of HWE-AAP(Wang, Liu, et al., 2024). At a concentration of 500 μg/mL, NO release in the UHP-AAP-treated group decreased to approximately 40 μmol/mL, with an inhibition rate exceeding 60%. At the same concentration, the HWE-AAP group still maintained a relatively high NO level (approximately 57 μmol/mL), with an inhibition rate of only about 43% (*p* < 0.05).

The regulatory effects of UHP-AAP and HWE-AAP on the secretion of pro-inflammatory cytokines IL-1β, IL-6, and TNF-α were consistent with the results for NO release (Figs. 2C–E). LPS stimulation upregulates the secretion levels of IL-1β, IL-6, and TNF-α in RAW264.7 macrophages, while both polysaccharides can dose-dependently reduce the expression and secretion of these cytokines(Yuan et al., 2022). At 500 μg/mL, UHP-AAP reduced IL-1β, IL-6, and TNF-α by 44%, 28%, and 39%, respectively, outperforming the corresponding HWE-AAP treatment group overall. UHP-AAP exhibited superior immunomodulatory capabilities while maintaining good biocompatibility. This synergistic advantage in structure and function provides an important molecular basis for its further integration with soy protein isolate to construct functional composite nanoparticles.

These functional characteristics reflect the potential for intermolecular interactions of polysaccharides, which determines their assembly behavior with proteins. The enhanced emulsifying properties and immunomodulatory activity of UHP-AAP are both intrinsically related to its molecular conformation and interaction capacity. The improved interfacial adsorption behavior reflects a stronger ability to interact with surrounding molecules, while the enhanced biological activity suggests a higher affinity toward biological interfaces such as cell membranes and receptors.

These characteristics collectively indicate that UHP-AAP possesses superior intermolecular interaction potential, which is critical for its subsequent assembly with proteins. Therefore, UHP-AAP may provide favorable structural characteristics for interaction with SPI and subsequent nanoparticle formation.

### 3.3 Effects of different factors on the zeta potential, particle size, and PDI of black fungus polysaccharide-soy protein isolate composite nanoparticles

**Fig. 3:**
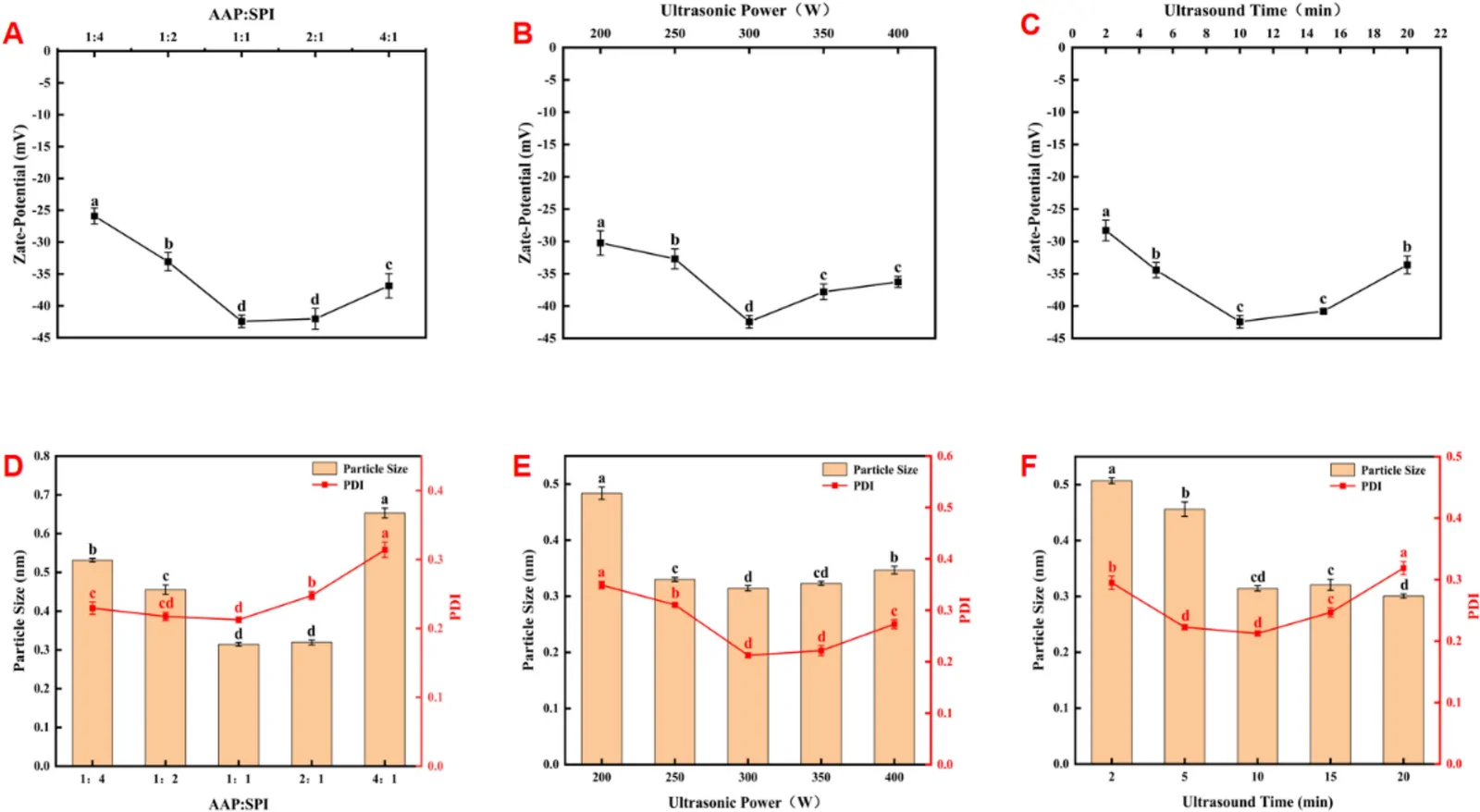
Zeta potential, particle size, and PDI of S-A NPs prepared by ultrasonication.

Auricularia auricula-judae polysaccharides (AAP) and soy protein isolate (SPI) formed a stable aqueous dispersion before ultrasonic treatment. Under ultrasonic treatment, the molecular chains are unfolded and activated, promoting intermolecular interactions including electrostatic attraction, hydrogen bonding, and hydrophobic interactions. Under optimized conditions, uniformly distributed, structurally stable nanoparticles with small particle sizes and high zeta potentials are formed, as shown in Fig. 3.

As shown in Fig. 3, the ratio of AAP in the system was gradually increased from 1:4 to 4:1, the absolute value of the zeta potential, particle size, and PDI of the composite nanoparticles all exhibited a distinct nonlinear trend. Among these, the 1:1 ratio demonstrated the most balanced physicochemical properties, with a zeta potential of −43 mV, the smallest particle size (318 nm), and the lowest PDI (approximately 0.2136). Absolute zeta potential values above 30 mV are considered sufficient to provide strong electrostatic repulsion and prevent particle aggregation. At a moderate ratio, the anionic properties of AAP, combined with the amphiphilic structure of SPI, resulted in a more balanced charge distribution(Li & Yu, 2020). This may improve intermolecular compatibility and reduce excessive aggregation, thereby constructing a more stable and uniform nanostructure. Under conditions of a low AAP ratio (1:4), SPI dominates the system, leading to insufficient interfacial charge density. This results in weak electrostatic repulsion between particles, making the system prone to aggregation. Conversely, when the AAP ratio is too high (4:1), both particle size and PDI increase. This phenomenon may be associated with excessive polysaccharide coverage and imbalance of intermolecular interactions. AAP and SPI, an amphiphilic protein, undergo electrostatic adsorption and hydrogen bonding at the interface(Lin et al., 2022). Excessive polysaccharide coverage may lead to increased steric hindrance and restricted interfacial rearrangement, which is detrimental to the formation of a stable structure (*p* < 0.05).

#### 3.3.1 Effect of ultrasonic power on S-A NPs formation

Ultrasonic power also influences the formation process of S-A nanoparticles. As the power increased from 200 W to 300 W, the absolute value of the system's zeta potential significantly increased and reached a peak, showing a positive correlation with system stability. Concurrently, particle size and PDI decreased to their lowest levels, indicating that the system formed the most stable dispersion state under these conditions. However, when the power was further increased, all parameters showed a certain degree of rebound, suggesting that excessive energy input may disrupt the already formed interfacial structure. Under low-power conditions, molecular dispersion and contact between AAP and SPI are insufficient, and interactions between polysaccharides and proteins are limited, making it difficult to form a uniform structure, resulting in larger particle sizes and uneven distribution. Under moderate power, however, molecular chain unfolding and mutual contact are effectively promoted, thereby facilitating the formation of stable nanostructures(Zou et al., 2021). Therefore, 300 W can be considered a relatively suitable ultrasonic power (*p* < 0.05).

#### 3.3.2 Effect of sonication time on S-A NPs

As shown in Figs. 3C and F, when the sonication time was extended from 2 min to 10 min, the absolute value of the zeta potential gradually increased and reached a maximum, while the particle size and PDI decreased to their lowest points. Further extension of the sonication time (to 20 min) resulted in a deterioration of these parameters. The core logic behind the time optimization lies in the cumulative effect of cavitation(Li et al., 2022). When the duration is too short (2 min), the polysaccharides and proteins in the system have not yet fully come into contact and bound. Prolonged mechanical action disrupts the formed AAP-SPI composite structure, promoting re-aggregation of molecular assemblies, and may also cause slight breakage of the polysaccharide molecular chains, leading to a decrease in system stability. Therefore, 10 min was determined to be the optimal sonication time (*p* < 0.05).

### 3.4 Infrared spectra of black fungus polysaccharide–soybean isolated protein composite nanoparticles

**Fig. 4:**
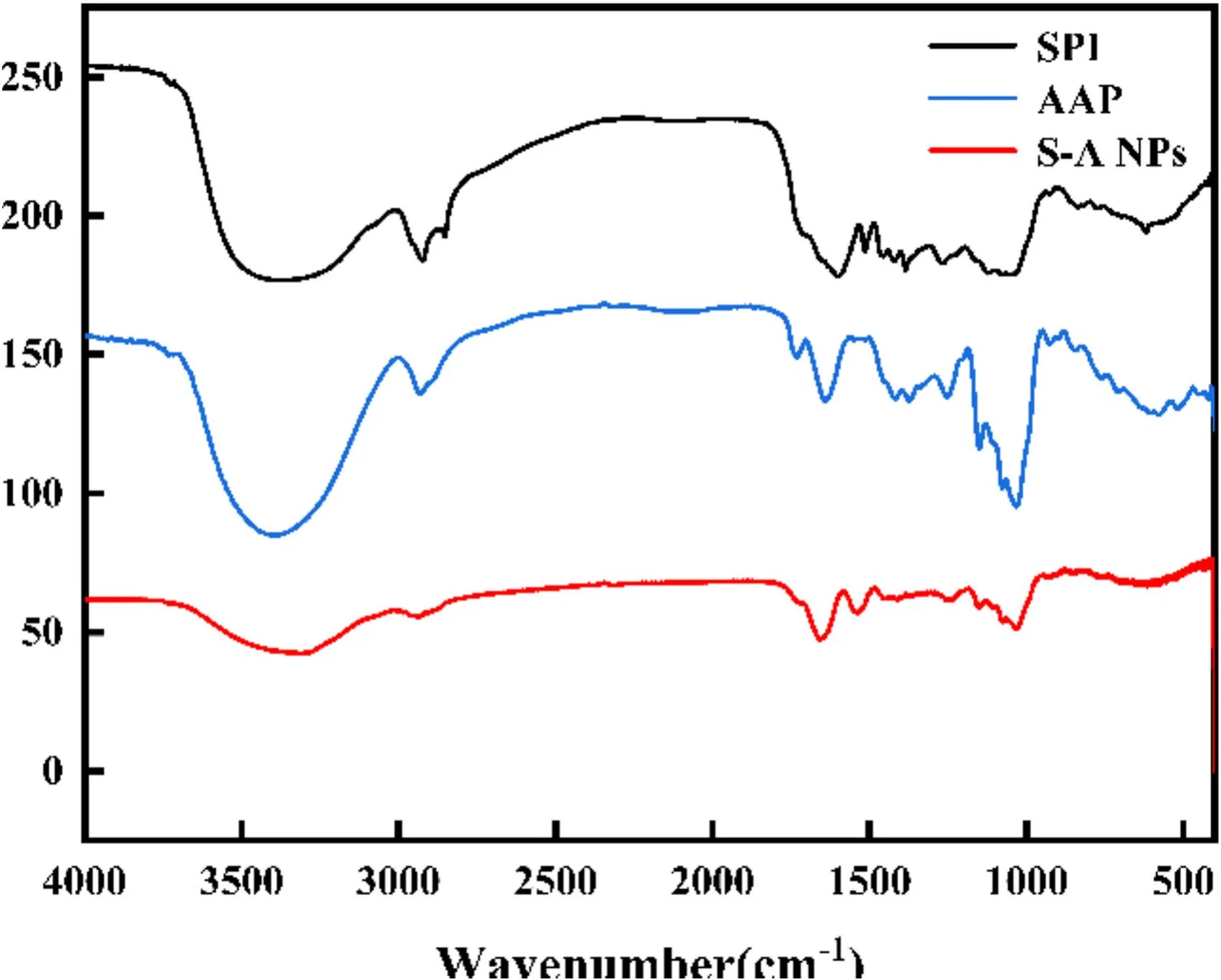
Comparison of FT-IR spectra of AAP, SPI, and AAP–SPI composite nanoparticles (S-A NPs).

Fourier transform infrared spectroscopy (FT-IR) was used to analyze the structural characteristics and interactions of SPI, AAP, and their composite nanoparticles (S-A NPs)(Lin et al., 2022). The results are shown in Fig. 4. Compared to the individual components, the spectrum of S-A NPs is not a simple superposition but exhibits distinct peak shifts, changes in band shapes, and adjustments in absorption intensity, suggesting the occurrence of intermolecular interactions between AAP and SPI. In the 3000–3600 cm^−1^ region (O–H/N–H stretching vibration region), the characteristic absorption bands of SPI and AAP merged into a broader absorption peak after composite formation, with a slight red shift (approximately 3280 cm^−1^) and a decrease in peak intensity. This change is typically associated with enhanced intermolecular hydrogen bonding, suggesting that a new hydrogen bond network may have formed between hydroxyl groups of AAP and polar groups (e.g., amide groups) of SPI, thereby reflecting changes in hydrogen-bonding environments. In the 1500–1700 cm^−1^ region, the amide I band of SPI at approximately 1650 cm^−1^ exhibited changes in peak shape and intensity after complex formation, while the amide II band around 1540 cm^−1^ also showed spectral variations(Aslan Türker, 2024). The C=O stretching vibration derived from glucuronic acid residues in AAP may partially overlap with the amide I region. These changes suggest that the interaction between AAP and SPI may alter the local chemical environment of protein functional groups and promote molecular rearrangement during complex formation(Xie et al., 2024). The carboxyl groups of the polysaccharide may interact electrostatically with the positively charged regions of SPI, thereby altering the local hydrogen-bonding environment of the protein and potentially affecting the local protein conformation. In the 1000–1200 cm^−1^ region, the characteristic glycosidic bond peaks of AAP exhibited some shift and broadening after complexation, supporting the existence of interactions between the two. The FT-IR results suggest that AAP and SPI form a stable noncovalent complex primarily through hydrogen bonding and electrostatic interactions. These results suggest that AAP and SPI may associate through multiple non-covalent interactions, including hydrogen bonding and electrostatic interactions, rather than covalent cross-linking(Cheng et al., 2024; Zhao et al., 2025).

### 3.5 X-ray diffraction analysis (XRD)

**Fig. 5:**
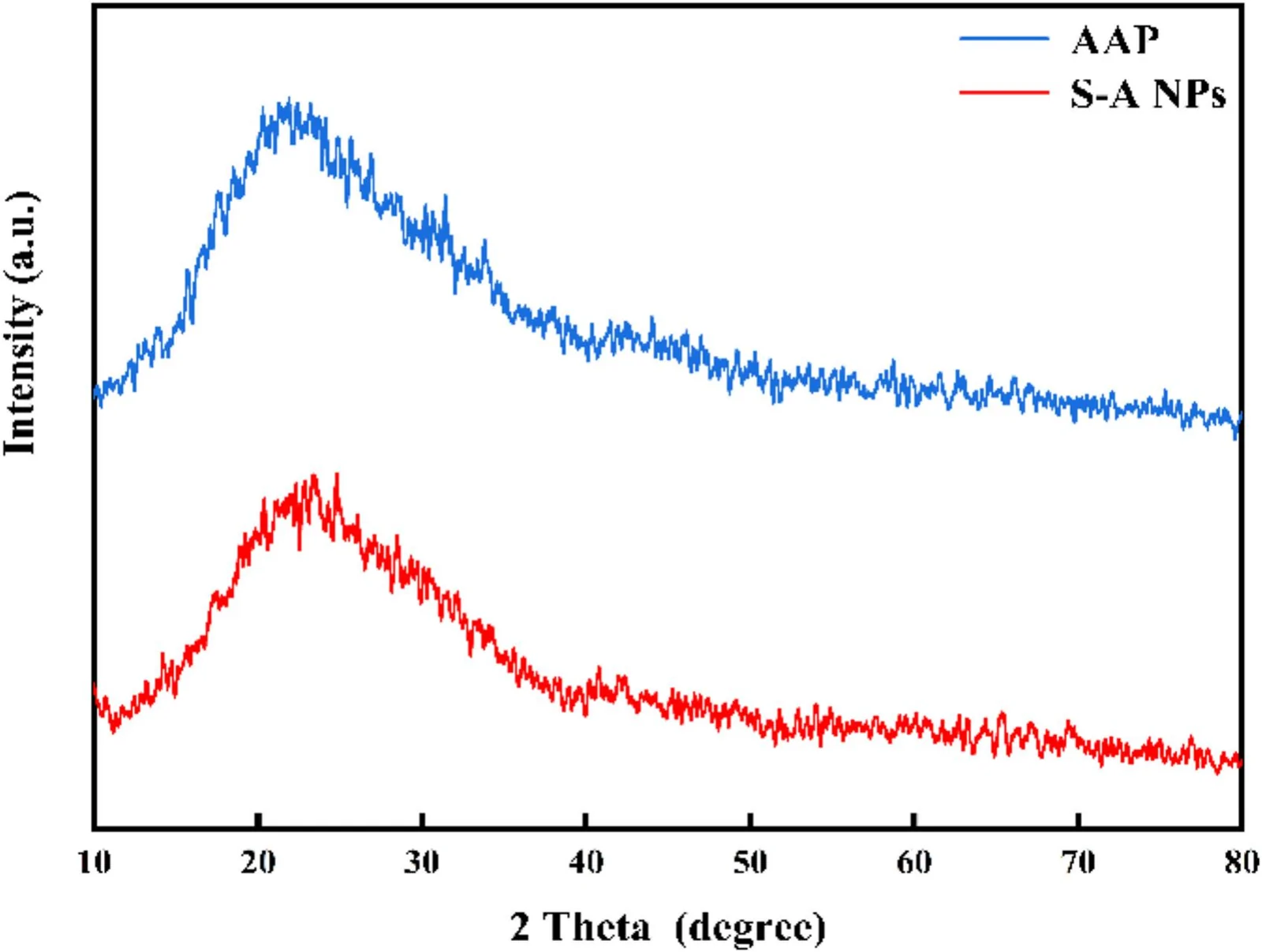
Comparison of XRD patterns for black fungus polysaccharides and black fungus polysaccharide–soybean isolate protein composite nanoparticles.

The results are shown in Fig. 5. Both samples exhibited a broad diffraction peak at 2θ ≈ 22°, indicating a predominantly amorphous structure(Yan et al., 2023). After complexation, the peak intensity decreased, and the peak becomes broader, suggesting a reduction in the degree of ordered arrangement and a decrease in crystallinity. This change is typically associated with intermolecular interactions between polysaccharides and proteins. During the complexation process, SPI molecular chains can embed or entangle themselves between AAP polysaccharide chains, forming a stable complex network through hydrogen bonding, electrostatic interactions, and hydrophobic interactions. This disrupts the local ordered arrangement of the original chain segments, transforming the system into a more structurally uniform amorphous network. No new sharp crystalline diffraction peaks were observed in the diffraction patterns of S-A NPs, indicating that no new crystalline structures were formed during the complexation process and that no significant crystalline rearrangement occurred. This further demonstrates that the system relies primarily on non-covalent interactions for assembly. The hydrogen bonds and electrostatic interactions formed between AAP and SPI disrupt the regular stacking of polymer chains at the molecular level, thereby reducing the overall crystallinity.

### 3.6 Determination of the thermal stability of black fungus polysaccharide–soybean isolated protein composite nanoparticles

**Fig. 6:**
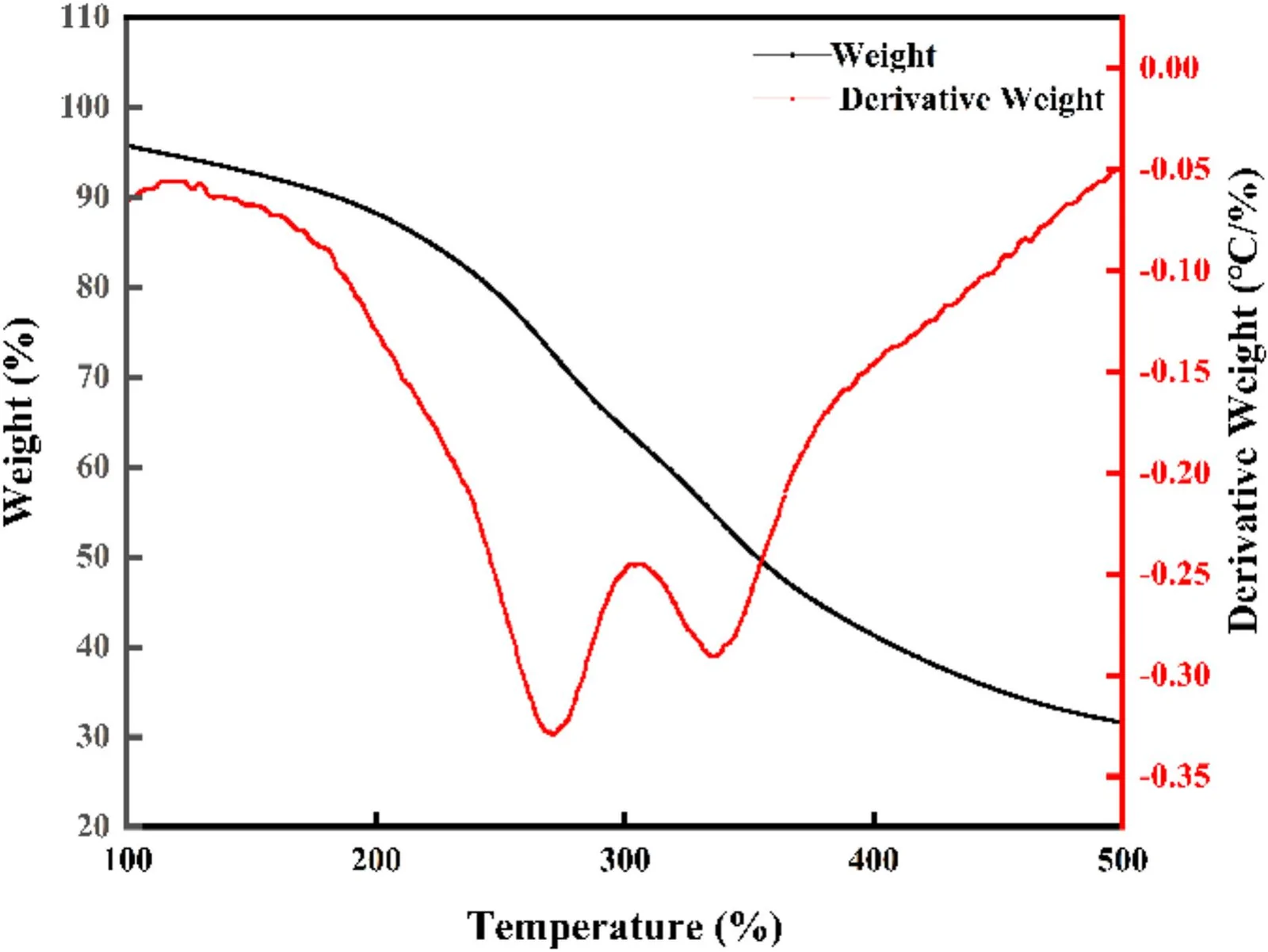
Thermogravimetric curve of AAP–SPI composite nanoparticles (S-A NPs).

The thermogravimetric analysis results reveal the thermal stability characteristics of the AAP–SPI composite nanoparticles from a thermal behavior perspective; their thermal degradation process is closely related to the molecular structures and interactions of the polysaccharides and proteins. The results are shown in Fig. 6. The main thermal degradation stages of the composite system are concentrated between 200 and 350 °C(Wang et al., 2018). The degradation temperature of the composite nanoparticles shifted toward higher temperatures compared with the individual components, indicating that the formation of the composite structure contributes to enhancing the thermal stability of the system to some extent. This phenomenon may be associated with the formation of intermolecular interactions between AAP and SPI. The hydroxyl groups on the AAP molecular chains can form a hydrogen bond network with the amide and carboxyl groups of SPI, accompanied by electrostatic interactions, thereby enhancing intermolecular bonding strength, improving structural stability, and delaying the onset of thermal degradation. The composite nanoparticles still retained a residual carbon content of approximately 32% at high temperatures (500 °C), indicating relatively high carbonization capacity during thermal decomposition. A higher residual carbon content generally indicates enhanced carbonization capacity and greater thermal resistance of the molecular structure (Chen et al., 2019). The increased thermal stability of AAP–SPI composite nanoparticles may be attributed to the formation of a strengthened intermolecular network through non-covalent interactions between AAP and SPI, including hydrogen bonding and electrostatic interactions. These interactions may restrict molecular mobility and delay thermal degradation during heating, resulting in a higher degradation temperature and residual carbon content compared with the individual components. The improved thermal resistance of AAP–SPI nanoparticles suggests their potential tolerance toward moderate thermal processing conditions commonly encountered in food-related applications.

### 3.7 Microstructural analysis (SEM) of black fungus polysaccharide–soybean isolated protein composite nanoparticles

**Fig. 7:**
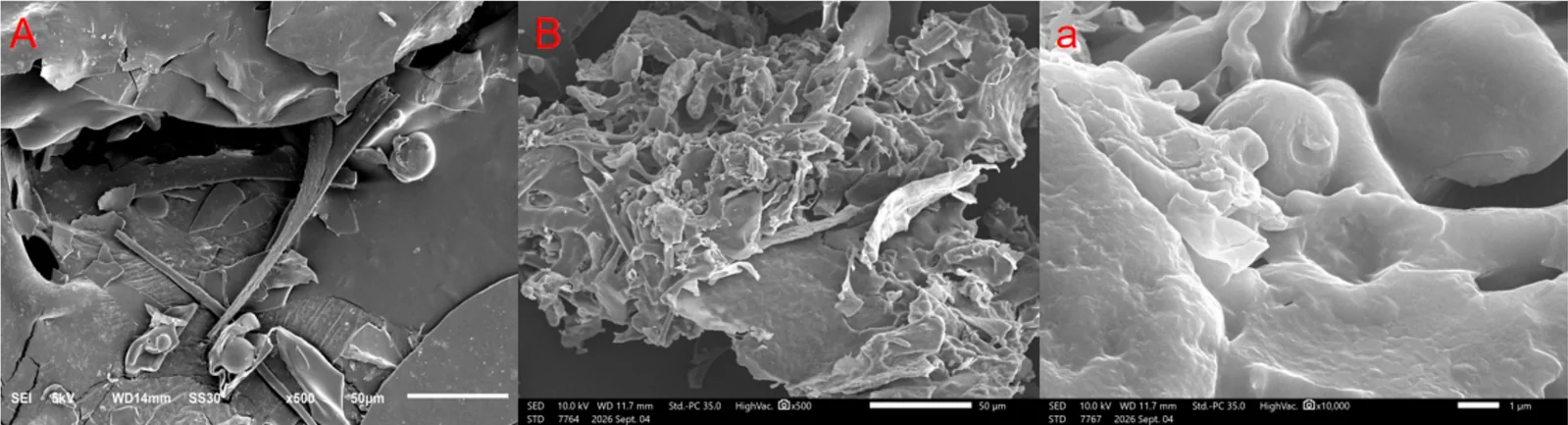
SEM images of AAP (A) and AAP–SPI composite nanoparticles (S-A NPs) (B—C).

The SEM micrographs of AAP and AAP-SPI composite nanoparticles (S-A NPs) are shown in Fig. 7. Native AAP exhibited large, compact, sheet-like stacked structures with intact lamellar morphology (Fig. A). After complexation with SPI, the original large-scale lamellar architecture of AAP was disrupted, and the samples turned into loose, porous and fragmented flocculent aggregates (Fig. B) (Albano et al., 2018; Yu, Pang, et al., 2024). Under high-magnification imaging (Fig. 7C), the rough, wrinkled surface and nanoscale protrusions of the composite particles further validate the nanoscale characteristics of the composite system (Xu et al., 2021), fully corroborating the previously reported results on particle size and zeta potential, this loose network structure not only increases the specific surface area of the particles, providing more sites for the exposure of active groups, but also enhances the system's aqueous dispersion stability through steric hindrance, thereby preventing particle aggregation.

## 4 Conclusion

This study demonstrates that ultra-high-pressure-assisted extraction (UHP) provides an effective strategy for regulating the structure and functionality of black fungus polysaccharides (AAP). Compared with conventional hot water extraction (HWE), UHP treatment improved the emulsifying performance and in vitro anti-inflammatory activity of AAP, which further facilitated its self-assembly with soy protein isolate (SPI). The optimized ultrasound-assisted AAP/SPI system (AAP:SPI = 1:1, 300 W, 10 min) formed stable composite nanoparticles through multiple non-covalent interactions, including hydrogen bonding and electrostatic attraction, resulting in improved thermal stability and colloidal properties. These findings reveal that extraction-induced structural modulation of polysaccharides can determine their subsequent assembly behavior, establishing a structure–function–assembly relationship for polysaccharide–protein systems. Although AAP–SPI nanoparticles show potential as functional food delivery carriers, further investigations involving in vivo validation, long-term stability, gastrointestinal behavior, and scalable processing are required. Future development of polysaccharide–protein-based delivery systems should focus on sustainable polymers, food-compatible additives, green processing technologies, and comprehensive sustainability evaluation.

## CRediT authorship contribution statement

**Qiong Wu:** Writing – original draft, Methodology, Investigation, Conceptualization. **Jiaxiu Wei:** Writing – original draft, Methodology, Investigation, Conceptualization. **Shuang Li:** Writing – original draft, Investigation. **JunXiao Du:** Writing – original draft. **ZhiTong Cai:** Writing – original draft. **TianQi Zhang:** Writing – original draft. **YongGang Dai:** Writing – review & editing, Resources, Methodology, Conceptualization.

## Fundings

This study was financially supported by the College of Food Science and Engineering, Changchun University, 10.13039/501100011845Jilin Academy of Agricultural Sciences, and the 10.13039/501100003807Jilin Province Science and Technology Development Program Project (20260102168JC).

## Declaration of competing interest

The authors declare that they have no known competing financial interests or personal relationships that could have appeared to influence the work reported in this paper.

## Data Availability

Data will be made available on request.
